# Factors associated with non-adherence to scheduled medical follow-up appointments among Cameroonian children requiring HIV care: a case-control analysis of the usual-care group in the MORE CARE trial

**DOI:** 10.1186/2049-9957-3-44

**Published:** 2014-12-03

**Authors:** Jean Joel R Bigna, Jean Jacques N Noubiap, Claudia S Plottel, Charles Kouanfack, Sinata Koulla-Shiro

**Affiliations:** Faculty of Medicine and Biomedical Sciences, University of Yaoundé 1, P.O. Box 1364, Yaoundé, Cameroon; Faculty of Medicine, University of Montpellier 1, Montpellier, France; Preventing Mother to Child Transmission Unit, Goulfey District Hospital, Goulfey, Cameroon; Internal Medicine Unit, Edéa Regional Hospital, Edéa, Cameroon; Department of Medicine, New York University Langone Medical Center, New York, USA; Accredited Treatment Centre, Yaoundé Central Hospital, Yaoundé, Cameroon; Infectious Diseases Unit, Yaoundé Central Hospital, Yaoundé, Cameroon

**Keywords:** MORE CARE, Lost to follow-up (LTFU), Appointment, Adherence, Children, Cameroon, HIV, AIDS, Preventing mother-to-child transmission (PMTCT), Missed scheduled medical appointment

## Abstract

**Background:**

A better understanding of why HIV-exposed/infected children fail to attend their scheduled follow-up medical appointments for HIV-related care would allow for interventions to enhance the delivery of care. The aim of this study was to determine characteristics of the caregiver-child dyad (CCD) associated with children’s non-adherence to scheduled follow-up medical appointments in HIV programs in Cameroon.

**Methods:**

We conducted a case-control analysis of the usual-care group of CCDs from the MORE CARE trial, in which the effect of mobile phone reminders for HIV-exposed/infected children in attending follow-up appointments was assessed from January to March 2013. For this study, the absence of a child at their appointment was considered a case and the presence of a child at their appointment was defined as a control. We used three multivariate binary logistic regression analyses. The best-fit model was the one which had the smallest chi-square value with the *Hosmer-Lemeshow* test (HLχ²). Magnitudes of associations were expressed by odds ratio (OR), with a *p*-value <0.05 considered as statistically significant.

**Results:**

We included 30 cases and 31 controls. Our best-fit model which considered the sex of the adults and children separately (HL χ²=3.5) showed that missing scheduled medical appointments was associated with: lack of formal education of the caregiver (OR 29.1, 95% CI 1.1–777.0; *p*=0.044), prolonged time to the next appointment/follow-up (OR [1 week increase] 1.4, 95% CI 1.03–2.0; *p*=0.032), and being a female child (OR 5.2, 95% CI 1.2–23.1; *p*=0.032). One model (HLχ²=10.5) revealed that woman-boy pairs adhered less to medical appointments compared to woman-girl pairs (OR 4.9, 95% CI 1.05–22.9; *p*=0.044). Another model (HLχ²=11.1) revealed that man-boy pairs were more likely to attend appointments compared to woman-girl pairs (OR 0.23, 95% CI 0.06–0.93; *p*=0.039). There were no statistical associations for the ages of the children or the caregivers, the study sites, or the HIV status (confirmed vs. suspected) of the children.

**Conclusion:**

The profile of children who would not attend follow-up medical appointments in an HIV program was: a female, with a caregiver who has had no formal education, and with a longer follow-up appointment interval. There is a possibility that female children are favored by female caregivers and that male children are favored by male caregivers when they come to medical care.

**Electronic supplementary material:**

The online version of this article (doi:10.1186/2049-9957-3-44) contains supplementary material, which is available to authorized users.

## Multilingual abstracts

Please see Additional file 1 for translations of the abstract into the six official working languages of the United Nations.

## Background

Only 34% of the 28.6 million people meeting criteria for antiretroviral therapy (ART) were successfully treated for human immunodeficiency virus (HIV) in low- and middle-income countries in 2013 [1]. In Cameroon, rates of ART coverage and of co-trimoxazole prophylaxis in children born to HIV positive mothers in 2011 remains low (9.2% and 5.4%, respectively), and shows a decline of more than 7% and 10%, respectively, compared to 2010 [2]. The low rate of treatment implementation reflects in part ineffective follow-up and monitoring of mother-child pairs in HIV programs. In 2011, only 65% of eligible children born to HIV-infected mothers were actually seen at the recommended six week post-partum visit for HIV-related care [2]. Compliance with medical visits—a component of the ART adherence indicator—can lead to a virological response to ART, occurrence of drug resistance, and mortality among people living with HIV [3, 4]. Mortality in HIV-infected infants has significantly decreased in the era of effective ART [5–7].

In Sub-Saharan Africa, the risk of loss to follow-up (LTFU) of children in an HIV program was 2.8% at six months, 4.6% at one year, and 8.4% at two years after introduction in the program [8]. Adherence to medical appointments to treat HIV is especially important, as clinic visits after initiation of highly active ART is an independent predictor for long-term clinical progress in HIV patients [9]. As detailed in a qualitative study, several reasons may explain why children requiring HIV treatment do not attend their follow-up appointments. Contributors to non-adherence of medical visits include intrapersonal factors (the cost of transportation, food availability, time constraints due to concomitant work, fear of disclosure of HIV status for mother and child, parental perception that the child is healthy, and personal religious beliefs); interpersonal factors (male partner nonparticipation, familial stigmatization and conflicts); community factors (cultural norms, changing community dynamics, and perceived stigma); and factors related to the healthcare system (the clinic’s location, lack of patient-centered care, delays at the clinic, and the scheduling of appointments at different times for mother and child) [10]. Outcomes from a study on LTFU and related factors in preventing mother-to-child transmission (PMTCT) of HIV can be used to improve this situation [11].

Much work remains to be done to better understand why caregivers fail to bring their children to medical appointments. In addition—to the best of our knowledge—no study has ever been conducted in Cameroon with the aim of assessing the factors associated with missed medical appointments in HIV-exposed and infected children. The aim of this investigation was to therefore determine the factors associated with HIV-exposed and infected children’s non-adherence to scheduled medical appointments in Cameroon. Using a case-control design, the factors assessed were (*i*) the age of the caregivers and children, (*ii*) sex of the caregivers and children, (*iii*) HIV status of the children (*iv*) the caregivers’ highest level of education, (*v*) the time interval between scheduled appointments, and (*vi*) the place of residence. The results of this study could inform policymakers and clinical staff on how to implement programs and interventions that address LTFU, thus decreasing its rate in HIV-infected and HIV-exposed children, and leading to reduced morbidity and mortality.

## Methods

### Study design

We conducted a case-control data analysis of all the participants randomly allocated to the usual-care (control) arm of the MORE CARE trial. The trial’s study design has been described elsewhere [12, 13]. The aim of this multicenter, blinded, factorial, randomized controlled study was to determine the most effective and efficient methods to increase the attendance of HIV-exposed and HIV-infected children at their medical appointments in Sub-Saharan Africa, by comparing various appointment reminders—a phone call, a text message, or both—with no reminder (usual standard of care) [12, 13]. The appointment reminder method has been described in previous studies [12, 13]. Participants in the text message group were sent a text message reminder two days prior to their scheduled appointment, and those in the call group were called two days prior to their scheduled appointment. Participants in the text message plus call group were sent a text message reminder three days before their scheduled appointment and were called two days prior to their appointment. For this investigation, a child’s absence at their follow-up appointment was defined as a case and a child’s presence was considered a control. We then studied the specific baseline characteristics of why an appointment might have been missed.

### Settings

In 2012, HIV prevalence in Cameroon was 4.5% among adults aged 15–49 years. The percentage of infants born to HIV-positive women receiving a virological test for HIV within two months of birth was 46%. The number of new HIV infections among children declined from 9,700 in 2001 to 5,800 in 2012 [14]. The proportion of infants with HIV positive results by polymerase chain reaction (PRC) analysis on dried blood spots was 12.3% [15]. The estimated percentage of pregnant women living with HIV who received ART for preventing mother-to-child-transmission (PMTCT) was 64% [14]. In a 2013 study performed in three settings in Cameroon, it was found that half (51%) of the children in HIV programs attended their scheduled medical appointments [13].

Participants in the MORE CARE trial were enrolled in an urban, a semi-urban, and a rural setting, respectively, at the Essos National Hospital Insurance Fund, Kousséri Annex Regional Hospital (KARH), and the Goulfey District Hospital (GDH), as detailed previously [12, 13]. The Essos National Hospital Insurance Fund, the KARH, and the GDH are primary, secondary, and tertiary healthcare settings, respectively. In Cameroon, there are three types of HIV clinics, at different hospital levels. In both the Essos National Hospital Insurance Fund and the KAHR, there is an accredited treatment center (ACT) (including a PMTCT clinic), which is the highest quality type of HIV clinic in Cameroon. The capacity of the ACT in the Essos National Hospital Insurance Fund is greater than that at the KAHR in terms of the number of employees, the space allocated to the HIV clinic, and the infrastructure. In the GDH, there is only a PMTCT clinic.

### Participants

The MORE CARE study enrolled caregiver-child dyads (CCDs). The present study includes all CCDs (adults aged 18 years or older who accompanied HIV-exposed or HIV-infected children, and the child) randomized to the MORE CARE trial control group (usual standard of care). Unlike the three other intervention arms, the usual standard of care cohort received no mobile phone reminder [12, 13]. For this analysis, cases are defined as all the CCDs (adult-child pairs) who were absent at the child’s follow-up scheduled medical appointment. Controls were all the CCDs pairs present at the child’s appointment. No matching has been performed.

### Variables

Nurses at the reception desk at each treatment center noted whether a child attended their appointment or not. We assessed the impact of the following factors on a CCD’s non-adherence to the child’s scheduled follow-up appointment (absence):

- Demographic data: ages and sex of the child and the caregiver, highest level of the caregiver’s education;
- HIV status: the HIV status of the child was either unknown or positive. Children with an unknown HIV status needed (and were scheduled) an appointment to find out the results of diagnostic testing. Children already infected with HIV were scheduled appointments for initiation and follow-up of ART therapy, for monitoring by a physician, and/or for counseling of the caregiver, as well as others;
- Settings: urban, rural, and semi-urban areas;
- Duration of time to the next scheduled medical appointment.

### Statistical analysis

Continuous variables were described using their mean and standard deviation (SD), and categorical variables using their frequencies and percentages. The χ^2^ test or Fisher’s exact test, when appropriate, were used to compare categorical variables between cases and controls, and the Student’s t-test was used to compare continuous variables. All variables assessed as factors in the bivariate analysis were integrated in the multivariate analysis using three models. In these three models, all variables were integrated but models differed in the analysis of the sexes of the adults and children.

In the first model, we integrated adult-child pairs based on sex, considering the adult and child as one entity. In the second model, we integrated the adult and child separately based on sex. This modeling permitted us to take each adult and child as a separate entity. In the third model, we looked at the interaction between the sex of the adult and the child. This model measured the dynamic interaction between the caregivers and children based on sex. The Hosmer-Lemeshow test, a statistical test for goodness-of-fit, was used to determine which regression model was the best. The best-fit model was the one which had the smallest chi-square value with the Hosmer-Lemeshow test.

A multivariate analysis was performed by the binary logistic regression model excluding non-significant variable (p >0.10). A *p* value <0.05 was considered as statistically significant. A null hypothesis of no difference was rejected for p < 0.05, or, equivalently, if the 95% confidence interval (CI) of odds ratio (OR) estimates excluded 1. Analyses were performed using the Statistical Package for Social Sciences (SPSS) for Windows version 20.0 (IBM Corp. Released 2011. IBM SPSS Statistics for Windows, Version 20.0. Armonk, NY: IBM Corp).

### Ethical considerations

The study was approved by the Faculty of Medicine and Biomedical Sciences Review Board at the University of Yaoundé 1, in Cameroon. We obtained ethical clearance from the National Research Ethics Committee for Human Health in Cameroon (Ethical approval No. 2013/03/232/L/CNERSH/SP). All caregivers included in the study provided written consent.

## Results

### Study population

In total, 61 adult-child pairs were potentially eligible (31 CCD cases and 30 CCD controls, therefore 1:1 ratio). All were confirmed eligible, and were included and analyzed.

### Descriptive analysis of participants

For all participants (cases and controls), the mean age of children was 1.6 (SD 2.4) years, and the mean age of caregivers was 42.6 (SD 12.6) years. There were 30 male caregivers (49.2%) and 8 male children (13.1%) among the participants, and the mean interval of time until the next scheduled medical appointment was 5.8 (SD 2.8) weeks. The HIV status was unknown in 34 children (55.7%) and positive in 27 (44.3%). In terms of the study sites, 17 (27.9%), 36 (59.0%), and 8 (13.1%) children came from rural, semi-urban, and urban areas, respectively. Out of the 61 caregivers, 37 (60.7%), 14 (23.0%), 6 (9.8%), and 4 (6.6%) had no formal, primary, secondary, and tertiary level of education, respectively.

### Bivariate analysis

The bivariate analysis is reported in Table 1. Cases and controls differed depending on the sex of the child—among the participants, more boys missed appointments (*p* =0.030)—and by the time period until the next scheduled appointment (this was longer among participants who missed their appointments) (*p* =0.033). There was no statistically significant difference between groups for the following variables: age (either children or caregivers), caregivers’ sex, adult-child sex, caregivers’ level of education, study sites, and children’s HIV status.

**Table 1:** **Bivariate analysis comparing cases (absence at appointment) and controls (presence at appointment)**

Variables	Absent at appointment n =30	Present at appointment n =31	p
Children age (years), mean (SD)	1.3 (1.9)	2.0 (2.8)	.29
Caregivers age (years), mean (SD)	41.9 (13.2)	43.2 (12.1)	.70
Boys, n (%)	19 (63.3)	11 (35.5)	.030
Men, n (%)	5 (16.7)	3 (9.7)	.47
Sex of adult-child pairs, n (%)			.15
● Man-boy	3 (10.0)	1 (3.2)	
● Man-girl	2 (6.7)	2 (6.5)	
● Woman-boy	16 (53.3)	10 (32.3)	
● Woman-girl	9 (30.0)	18 (58.1)	
Caregivers’ level of education, n (%)			.46
● No formal	21 (70.0)	16 (51.6)	
● Primary	6 (20.0)	8 (25.8)	
● Secondary	2 (6.7)	4 (12.9)	
● University	1 (3.3)	3 (9.7)	
Study sites, n (%)			.37
● Rural	10 (33.3)	7 (22.6)	
● Semi-urban	15 (50.0)	21 (67.7)	
● Urban	5 (16.7)	3 (9.7)	
HIV status of children, n (%)			.16
● Unknown	14 (46.7)	20 (64.5)	
● Positive	16 (53.3)	11 (35.5)	
Time to appointment (weeks), mean (SD)	6.6 (2.8)	5.1 (2.6)	.033

IQR: interquartile range; SD: standard deviation; OR: odds ratio; CI: confidence interval.

### Multivariate logistic regression analysis

The multivariate analyses are presented in Table 2. In the first model, which integrated the sex of the adult-child pairs but not the individual sex of the children and adults, the factors associated with missing scheduled appointments were: woman-boy pair (OR 4.9; 95% CI 1.05–22.9; *p* =0.044) compared to woman-girl pair, caregiver not having had any formal education (OR 29.7; 95% CI 1.1–791.5; *p* =0.043) compared to university education, and time to the next appointment (OR [1 week increase] 1.4; 95% CI 1.03–2.0; *p* =0.031).

**Table 2:** **Multivariate regression analysis assessing factors for not attending a medical appointment**

	Model (1)		Model (2)		Model (3)	
Variables	***p***	OR (95% CI)	***p***	OR (95% CI)	***p***	OR (95% CI)
Children age, years (continuous)	.14	0.80 (0.59; 1.1)	.12	0.79 (0.59; 1.01)	.10	0.78 (0.58; 1.1)
Caregivers age, years (continuous)	.15	0.96 (0.91; 1.02)	.14	0.96 (0.91; 1.01)	.13	0.96 (0.90; 1.01)
Girls	-	Not used	.032	5.2 (1.2; 23.1)	-	Not used
Women	-	Not used	.40	0.41 (0.05; 3.3)	-	Not used
Sex of adult-child pair			-	Not used	-	Not used
● Man-boy	.22	22.4 (0.16; 3199.2)	-		-	
● Man-girl	.56	2.0 (0.19; 22.1)	-		-	
● Woman-boy	.044	4.9 (1.05; 22.9)	-		-	
● Woman-girl	.20	1.0	-		-	
Men – Boys interactions	-	Not used	-	Not used	.039	0.23 (0.06; 0.93)
Caregivers’ education level						
● No formal	.043	29.7 (1.1; 791.5)	.044	29.1 (1.1; 777.0)	.044	29.2 (1.1; 782.7)
● Primary	.23	6.9 (0.30; 161.6)	.23	6.8 (0.29; 158.3)	.21	7.5 (0.32; 176.4)
● Secondary	.73	2.3 (0.02; 248.6)	.55	3.4 (0.06, 176.3)	.33	6.2 (0.15; 247.8)
● Tertiary	.15	1.0	.14	1.0	.17	1.0
Study sites						
● Rural	.16	0.12 (0.006; 2.3)	.15	0.12 (.006; 2.2)	.13	0.10 (0.01; 2.0)
● Semi-urban	.12	0.09 (0.04; 1.8)	.11	0.09 (.004; 1.8)	.10	0.08 (0.004; 1.6)
● Urban	.30	1.0	.28	1.0	.25	1.0
Unknown HIV status of children	.08	0.28 (0.07; 1.2)	.08	0.27 (0.06; 1.1)		
Time to next appointment, weeks (continuous)	.031	1.4 (1.03; 2.0)	.032	1.4 (1.03; 2.0)	.036	1.4 (1.02; 2.0)
**Hosmer**-**Lemeshow test**						
Degree of freedom	8		8		8	
*p*-value	.235		.898		.199	
Chi-squared	10.5		3.5		11.1	

In the second model, which separately considered the sex of the children and adults but not of the adult-child pair, the factors associated with missing scheduled appointments were: female child (OR 5.2; 95% CI 1.2–23.1; *p* =0.032), caregiver not having had any formal education (OR 29.1; 95% CI 1.1–777.0; *p* =0.044) compared to university education, and time to the next appointment (OR [1 week increase] 1.4; 95% CI 1.03–2.0; *p* =0.032).

In the third model, which looked at the interaction between the sex of the child and the adult, the factors associated with missing scheduled appointments were: interaction between men and boys (OR 0.23; 95% CI 0.06–0.93; *p* =0.039), caregiver not having had any formal education (OR 29.2; 95% CI 1.1–782.7; *p* =0.044) compared to university education, and time to next appointment (OR [1 week increase] 1.4; 95% CI 1.02–2.0; *p* =0.036).

The Hosmer-Lemeshow test showed that the best regression model for our study was the second, in which the sex of the caregivers and the children were considered separately (chi-squared =3.5 < 10.5 [first model] and 11.1 [third model]).

## Discussion

This study showed that in a pediatric HIV program in Cameroon, a child is more likely to not attend their next scheduled medical appointment if that child is female, has a caregiver who has not had any formal education, and if there is a longer interval until the next scheduled appointment. There were no associations found between the child attending a follow-up HIV medical appointment and the child or caregiver’s age, the sex of the caregiver, residence, or knowledge of the child’s HIV status.

Our study found that the most significant determinant of not attending medical appointments was the caregiver’s lack of formal education. A lack of formal education could lead to a caregiver not truly understanding the importance of follow-up appointments. It is also possible that the language being used by physicians is too complex for caregivers with no formal education to understand. As demonstrated by Okoronkwo and colleagues, patients with no formal education in a HIV clinic in Nigeria were more likely to have poor communication skills [16]. The quality of patient-physician communication is therefore paramount [17]. A study in Kenya showed that a good perception of patient-physician communication by the patient in an HIV clinic is associated with a low likelihood of patients missing appointments [18]. Certain factors have been associated with better communication between patients and physicians at HIV clinics: longer visits, longer duration of the physician-patient relationship, and the patient being of the female sex [19].

In a study done in China measuring early missed visits to HIV clinics, unaccompanied women were more likely to not attend medical appointments [20]. It was also found in several studies that unaccompanied adult males were more likely to not attend medical appointments in resource-limited settings [21–25]. We did not find any study integrating adult-child pairs, or that showed an interaction between child and caregivers based on sex. In this study, boys accompanied by women adhered less to medical appointments compared to girls accompanied by women. In addition, our best-fit model, which considered the sex of the caregiver and child separately, found that male children were more likely to be brought to medical appointments. In the third model, a good relationship was observed between men and boys compared to women and girls, in terms of compliance with medical appointments. The findings raise several important hypothesis-generating questions which require further study. In the context of HIV care: Why is a male child favored over a female child in terms of follow-up appointments? Why do female caregivers favor female children in attending appointments? Why is the sex congruence between male caregivers and male children better than that of female caregivers and female children in terms of follow-up? One hypothesis is that male children are favored by male caregivers and female children are favored by female caregivers when it comes to medical care in Sub-Saharan Africa.

The third factor concerned the time interval to the next scheduled appointment. The longer the time period to the next appointment, the higher the failure rate of keeping appointments. This is consistent with forgetfulness over time as shown by other studies: in adult patients living with HIV [16, 26] and in other non-HIV populations [27–35].

Therefore, health professionals responsible for scheduling children’s medical appointments should pay extra attention to caregivers who haven’t had any formal education, and to female adults accompanying female children. Also, appointments should be scheduled as soon as the need for one arises so as to lessen the chance of forgetting. In addition, we advocate that a reminder be sent via mobile phones as demonstrated in the MORE trial [13] and in other studies [36–44]. Good communication between patients and physicians [17] involves several factors including longer visits, longer duration of the physician-patient relationship, and the sex of the patient and the physician [19].

Our study had limitations. A case-control design is not the best method to measure the direct effect of variables on attending a medical appointment. It is likely that the quality of care differs from one site to another, therefore the impact of the area (urban, semi-urban, and rural) was not only related to the socioeconomic level but also to the quality of care. Finally, a larger sample of caregiver-child dyads might uncover additional significant associations. The associations we have found between children’s missed appointments for HIV care and the children’s sex, the caregiver’s level of education, and the time to the next appointment suggest that interventions can be implemented at each site, and also call for further research. Interventions could include better-directed counseling for caregivers; assessment of the caregiver’s educational level, with information exchange tailored to that level; and shortening the time to the next appointment. Despite these shortcomings, this study permits us to define one of the profiles of children not attending scheduled medical appointments at HIV clinics.

## Conclusions

This study examined 61 Cameroonian caregiver-child dyads, in which the child needed follow-up medical care for HIV. The outcome of interest was a child’s non-attendance at their next appointment. Missed follow-up was associated with the sex of the child (female), the education level of the caregiver (no formal education), and the time to the next appointment (longer). Other factors, such as the age of the child, the age and the sex of the caregiver, the child’s HIV status (confirmed vs. at risk), and the geographic location, had no demonstrable correlation with missed appointments. The findings point to specific targets for potential interventions to enhance the follow-up care of Cameroonian children with HIV. We therefore recommend that clinicians use basic language (if possible, local dialect) when communicating with their patients or caregivers of their patients, especially those who have not had any formal education. Counseling for caregivers accompanying a female child must also be increased. As much as logistically possible, the health professional must aim to reduce the time between appointments and/or implement a method of appointment reminders.

## Electronic supplementary material

Additional file 1: **Multilingual abstracts in the six official working languages of the United Nations.** (PDF 369 KB)

## Abbreviations

ACT: Accredited treatment center
ART: Antiretroviral therapy, also antiretroviral treatment
CCD: Caregiver-child dyad
CI: Confidence interval
df: degree of freedom
HIV: Human immunodeficiency virus
IQR: Interquartile range
LTFU: Lost (or loss) to follow-up
MORE CARE: Mobile Reminders for Cameroonian Children Requiring HIV Treatment
OR: Odds ratio
PMTCT: Preventing mother-to-child transmission.
