# Supplementary material for: Factors associated with non-adherence to scheduled medical follow-up appointments among Cameroonian children requiring HIV care: a case-control analysis of the usual-care group in the MORE CARE trial
Source: Infect Dis Poverty. 2014 Dec 3;3:44. doi: 10.1186/2049-9957-3-44 (PMC4322435; doi:10.1186/2049-9957-3-44)
Supplement: Supplementary file 1 — Additional file 1: Multilingual abstracts in the six official working languages of the United Nations. (PDF 369 KB) [file 40249_2014_85_MOESM1_ESM.pdf]

Translation of the abstract into the six official working languages of the United Nations

العوامل المرتبطة بعدم المواظبة على حضور مواعيد المتابعة الطبية المقررة بين الأطفال الكاميرونيين الذين تتطلب حالاتهم رعاية لإصابتهم بفيروس نقص المناعة البشرية: تحليل الحالات والشواهد لمجموعة الرعاية المعتادة في تجربة رقابة الأجهزة المزامنة ومرضى القلب MORE CARE

جان جويل ر بينيا، جان جاك ن. نويباب، كلوديا س. بلوتل، تشارلز كوانفاك، سيناثا كولا-شيرو

الخلاصة

خلفية: الفهم الأفضل لسبب عدم مواظبة الأطفال المعرضين للإصابة بفيروس نقص المناعة البشرية والمصابين بفيروس نقص المناعة البشرية في الالتزام بالحضور في المواعيد المقررة للمتابعة أثناء حصولهم على الرعاية الطبية من تأثيرات نقص المناعة البشرية من شأنه أن يسمح بتقديم الرعاية بصورة أفضل. وكان الهدف من هذه الدراسة تحديد خصائص الرعاية للأطفال (CCD) المرتبطة بعدم التزام الأطفال بالحضور في المواعيد المقررة للمتابعة في إطار برامج رعاية مرضى فيروس نقص المناعة البشرية في الكاميرون.

الأساليب: أجرينا تحليل للحالات والشواهد لمجموعة الرعاية المعتادة (المجموعة الضابطة) لمجموعة الرعاية المعتادة في تجربة رقابة الأجهزة المزامنة ومرضى القلب، حيث تم تقييم تأثير تذكير الاطفال المصابين / المعرضين للإصابة بفيروس نقص المناعة البشرية من خلال الهاتف المحمول بحضور مواعيد المتابعة في ثلاثة إعدادات (المناطق الحضرية وشبه الحضرية والريفية) من يناير إلى مارس 2013. في هذه الدراسة، اعتبر غير المواظبين على الحضور في مواعيدهم المجموعة التجريبية والأطفال المواظبين على الحضور في مواعيدهم المجموعة الضابطة. واستخدمنا ثلاثة تحليلات لوجستية ثنائية الانحدار متعددة المتغيرات. كان أفضل نموذج في التناسب ذلك الذي كانت قيمة خي مربع فيه الأقل مع اختبار هوسمر- ليمشو (HL χ^2). تم التعبير عن مقادير الاقترانات من خلال نسبة الأرجحية (OR)، مع اعتبار القيمة P <0.05 ذات دلالة إحصائية.

النتائج: من بين 30 طفلا الذين لم يواظبوا على الحضور في مواعيدهم (المجموعة التجريبية) و31 الذين حضروا للمتابعة (المجموعة الضابطة)، كان متوسط عمر الأطفال 1.3 (الانحراف المعياري 1.9) و 2.0 (2.8) عاما (ع = 0.29)، على التوالي؛ وكان متوسط العمر لمقدمي الرعاية 41.9 (13.2) و 43.2 (12.1) عاما (ع = 0.70) على التوالي. أظهر نموذجنا الأفضل مناسبة الذي يدرس جنس البالغين والأطفال على حدة = χ^2 HL) (3.5أن عدم المواظبة على حضور المواعيد الطبية ارتبط بالتالي: افتقار مقدم الرعاية إلى التعليم الرسمي (OR 29.1، 95٪ CI 1-777،1،0؛ ع = 0.044)، طول الفترة بين مواعيد المتابعة (OR [الزيادة بمعدل أسبوع] 1.4، 2.0–1.03 CI 95%؛ p = 0.032)، كون المريضة طفلة (OR 5.2، 95٪ CI 1.2– 23.1 ، P = 0.032). كما بينت النماذج الإضافية وجود ارتباط بين الأطفال الإناث في رعاية الأطفال وعدم المواظبة في الحضور في المواعيد، بغض النظر عن كون مقدم الرعاية ذكرا أو أنثى. وأظهر أحد نماذج الانحدار (HL χ^2 = 10.5) أن الحالات التي يكون فيها المريض صبي ومقدم الرعاية الطبية امرأة أقل تقيدا بالمواعيد مقارنة مع الحالات التي يكون فيها المريض فتاة ومقدم الرعاية الطبية امرأة (OR 4.9، 95% CI 1.05–22.9؛ p = 0.044). نموذج آخر (HL χ^2 = 11.1) أظهر أن الحالات التي يكون فيها المريض صبي ومقدم الرعاية الطبية رجل كانت أكثر التزاما بالمواظبة على حضور المواعيد مقارنة مع الحالات التي يكون فيها المريض فتاة ومقدم الرعاية الطبية امرأة (OR 0.23، 95% CI 0.06–0.93؛ p = 0.039). لم تلاحظ اقترانات إحصائية بين الأعمار في الأطفال أو مقدمي الرعاية، ومواقع الدراسة، أو وضع فيروس نقص المناعة البشرية (الحالات المؤكدة مقابل الحالات المشتبه في إصابتها) في الأطفال.

الاستنتاج: في العينة الكاميرونية من الأطفال الذين يحتاجون للمتابعة أثناء حصولهم على الرعاية الطبية من تأثيرات فيروس نقص المناعة البشرية، كان توصيف الأطفال الذين لم يواظبوا على حضور المواعيد الطبية للمتابعة في برنامج فيروس نقص المناعة البشرية كالتالي: أنثى، مع مقدم رعاية لم يحصل على تعليم رسمي، ومع فترة متابعة أطول. وأظهرت الدراسة أيضا أن هناك احتمال أن مقدمي الرعاية الإناث يفضلن الأطفال الإناث بينما يفضل مقدمو الرعاية الرجال الأطفال الذكور عندما يتعلق الأمر بالرعاية الطبية.

Translated from English version into Arabic by Mahmoud Sami, through

TRANSLATORS
WITHOUT BORDERS

喀麦隆需要艾滋病关怀儿童不能按期完成医学随访的影响因素分析：一个多中心病例对照研究

Jean Joel R. Bigna, Jean Jacques N. Noubiap, Claudia S. Plottel, Charles Kouanfack, Sinata Koulla-Shiro

摘要

引言：更好地理解为什 HIV 感染/暴露儿童未能按期完成 HIV 相关关爱的医学随访，将有助于采取措施增加 HIV 关怀的执行。本研究的目的是了解喀麦隆 HIV 项目中儿童（看护者和儿童对子）是否能按期完成医学随访的主要影响因素。

方法：2013 年 1～3 月，我们在需要 HIV 关怀的儿童中开展了一个病例对照研究，这些儿童分别来自农村，城郊结合部和城区。在研究中未能按时完成随访的儿童作为病例组，能够按时完成随访的儿童作为对照组。使用多因素 Logistic 回归模型进行结果分析，*Hosmer-Lemeshow* 检验卡方值最小的作为最优拟合模型，用 *OR* 值大小来反映相关性，P<0.05 被认为有统计学意义。

结果：30 名未能按期参加随访的儿童作为病例组，31 名按时参加随访的儿童为对照组。病例组和对照组的平均年龄为分别为 1.3±1.9 岁和 2.0±2.8 岁，两组年龄差别无统计学意义（P=0.29）。看护者的平均年龄分别为 41.9±13.2 岁和 43.2±12.1 岁，两组年龄差别无统计学意义（P=0.70），成年人和儿童性别（HL χ^2=3.5）为变量的最优拟合模型显示，未能按期参加随访的影响因素有：看护者未接受过正规教育（OR 29.1, 95% CI 1.1–777.0; p = 0.044）、两次随访间隔时间长（(OR [增加 1 周] 1.4, 95% CI 1.03–2.0; p = 0.032)和女性儿童(OR 5.2, 95% CI 1.2–23.1; p = 0.032)。其他模型也显示，儿童性别为女性和未能按期随访有关，不管看护者是男性还是女性。一元回归模型(HL χ^2 = 10.5)显示，女性看护人和男孩结成的对子随访的依从性要比女性看护人和女孩结成的对子差(OR 4.9, 95% CI 1.05–22.9; p = 0.044)。另一个模型(HL χ^2 = 11.1)显示，男性看护人和男孩结成的对子要比女性看护人和女孩结成的对子更愿意参加医学随访。儿童年龄、看护者年龄、不同的研究现场以及儿童的 HIV 感染状态等变量无统计学意义。

结论：在喀麦隆需要 HIV 关怀随访的儿童中，导致他们不能按期参加 HIV 项目医学随访主要因素有女性、看护者未接受正规的教育和随访间隔时间太长。研究还显示有可能在参加医学随访时女性看护者通常优先考虑女孩，男性看护者通常优先考虑男孩。

Translated from English version into Chinese by Tian Li-guang, through

CHINESE CENTER FOR DISEASE CONTROL AND PREVENTION
NATIONAL INSTITUTE OF PARASITIC DISEASES

Facteurs associés à la non-observance des rendez-vous relatifs au suivi médical régulier chez des enfants camerounais ayant besoin de soins liés au VIH : analyse cas-témoins du groupe des soins habituels dans l'étude MORE CARE

Jean Joel R. Bigna, Jean Jacques N. Noubiap, Claudia S. Plottel, Charles Kouanfack, Sinata Koulla-Shiro

Résumé

Contexte : Une meilleure compréhension des raisons pour lesquelles les enfants exposés au VIH et infectés par le VIH ne viennent pas aux rendez-vous prévus de suivi médical relatif aux soins liés au VIH permettrait des interventions visant à améliorer les prestations de soins. Le but de cette étude était de déterminer les caractéristiques de la dyade soignant-enfant (DSE), associées à la non-observance par les enfants des rendez-vous planifiés de suivi médical dans les programmes VIH au Cameroun.

Méthodes : Nous avons mené une analyse cas-témoins du groupe des DSE sous soins habituels (contrôle) de l'étude MORE CARE, au cours de laquelle l'effet des rappels par téléphone mobile sur la présence aux rendez-vous de suivi des enfants exposés au VIH/infectés par le VIH a été évaluée dans trois contextes (urbain, semi-urbain et rural), de janvier à mars 2013. Pour cette étude, l'absence d'un enfant à son rendez-vous était considérée comme un cas et la présence d'un enfant à son rendez-vous était définie comme un contrôle. Nous avons utilisé trois analyses multivariées de régression logistique binaire. Le meilleur modèle était celui qui avait la plus petite valeur de chi-carré au test de *Hosmer-Lemeshow* (HL χ^2). Les forces de l'association étaient exprimées en risques relatifs (RR), avec une valeur de p <0,05 considérée comme statistiquement significative.

Résultats : Parmi les 30 enfants absents à leurs rendez-vous (cas) et les 31 enfants présents (contrôles), l'âge moyen des enfants était de 1,3 (écart-type 1,9) et 2,0 (2,8) ans (p = 0,29), respectivement ; l'âge moyen des soignants était de 41,9 (13,2) et 43,2 (12,1) ans (p = 0,70), respectivement. Notre meilleur modèle qui tenait compte du sexe des adultes et des enfants séparément (HL χ^2 = 3,5) montrait que l'absence aux rendez-vous médicaux réguliers était associée au manque d'éducation formelle du soignant (RR 29,1, IC à 95% 1,1-777,0 ; p = 0,044), à la longueur du délai jusqu'au prochain rendez-vous/suivi (RR [1 semaine de plus] 1,4, IC à 95% 1,03-2,0 ; p = 0,032), et au fait d'être un enfant de sexe féminin (RR 5.2, IC à 95% 1,2-23,1; p = 0,032). Une modélisation supplémentaire a également démontré une association entre les enfants de sexe féminin au sein de la DSE et les rendez-vous manqués, que le soignant soit masculin ou féminin. Un modèle de régression (HL χ^2 = 10,5) a révélé que les paires femme-garçon respectaient moins les rendez-vous médicaux que les paires femme-fille (RR 4,9, IC à 95% 1,05-22,9 ; p = 0,044). Un autre modèle (HL χ^2 = 11,1) a révélé que les paires homme-garçon étaient plus susceptibles de venir aux rendez-vous que les paires femme-fille (RR 0,23, IC à 95% 0,06-0,93 ; p = 0,039). Il n'a pas été observé d'associations statistiques entre les âges des enfants ou des soignants, les centres d'étude, ou le statut VIH (confirmé contre présumé) des enfants.

Conclusion : Dans notre échantillon camerounais d'enfants nécessitant des soins de suivi de VIH, le profil de l'enfant qui ne viendrait pas aux rendez-vous de suivi médical dans un programme VIH était : une fille, dont le soignant n'a pas eu d'éducation formelle, et ayant un plus long délai jusqu'au rendez-vous de suivi. L'étude a également montré qu'il existe une possibilité que les enfants de sexe féminin soient favorisés par les soignantes femmes et que les enfants de sexe masculin soient favorisés par les soignants hommes, quand il s'agit de soins médicaux.

Translated from English version into French by Jacek Sierakowski, through

TRANSLATORS
WITHOUT BORDERS

Факторы, связанные с несоблюдением назначенных посещений врача камерунскими детьми, нуждающимся в ВИЧ-уходе: изучение методом «случай-контроль» группы, получавшей стандартное лечение в исследовании MORE CARE

Жан Жоель Р. Бигна, Жан Жак Н. Нубьяп, Клаудиа С. Плоттель, Чарльз Куанфак, Сината Коулла-Широ

Аннотация

Краткое описание: Более глубокое понимание того, почему ВИЧ-инфицированные и подвергающиеся риску заражения дети не приходят на последующие приемы для лечения ВИЧ-инфекции, позволило бы принять меры по улучшению оказания медицинской помощи. Настоящее исследование было направлено на определение характеристик пары «ребенок и лицо, осуществляющее уход за ним, (CCD)», связанных с неявкой детей на назначенные им повторные посещения врача в рамках ВИЧ-программ в Камеруне.

Методы: Мы провели изучение методом «случай-контроль» пар CCD, входивших в группу, получавшую стандартное лечение, (контроль), в рамках исследования MORE CARE, в котором оценивались результаты звонков на мобильные телефоны с напоминанием ВИЧ-инфицированным/подвергающимся риску заражения детям о повторных посещениях врача. Изучение проводилось в трех средах (городская, полугородская и сельская) с января по март 2013 г. При этом непосещение ребенком врача в назначенное время считалось «случаем», а приход ребенка на прием считался «контролем». Мы использовали три модели многомерного бинарного логистического регрессионного анализа. Самой подходящей была модель с наименьшей величиной хи-квадрата с использованием теста *Хосмера-Лемешоу* (HL χ^2). Величины связей были выражены отношением шансов (OR) и считались статистически значимыми при значении $p<0{,}05$.

Результаты: Среди 30 детей, не пришедших к врачу, (случаи), и 31 ребенка, которые пришли на последующие приемы, (контроль), средний возраст детей составил 1,3 года (среднеквадратическое отклонение 1,9) и 2,0 года (2,8) (p = 0,29) соответственно; средний возраст лиц, осуществляющих уход, 41,9 года (13,2) и 43,2 года (12,1) (p = 0,70) соответственно. Наша наиболее оптимальная модель, которая учитывает пол взрослых и детей отдельно (HL χ^2 = 3,5), показала, что пропуск назначенных медицинских посещений был связан с: недостатком формального образования лица, осуществляющего уход, (OR 29,1; 95% CI 1,1–77,0; p = 0,044); большой продолжительностью периода времени до следующего посещения (OR [увеличение на 1 неделю] 1,4; 95% CI 1,03–2,0; p = 0,032), и женским полом ребенка (OR 5,2; 95% CI 1,2–23,1; p = 0,032). Дополнительное моделирование также продемонстрировало связь между детьми женского пола в парах CCD и пропущенными посещениями врача независимо от того, является ли лицо, осуществляющее уход, мужчиной или женщиной. Одна регрессионная модель (HL χ^2 = 10,5) выявила, что пары «женщина-мальчик» хуже соблюдали посещения врачей по сравнению с парами «женщина-девочка» (OR 4,9; 95% CI 1,05–22,9; p = 0,044). Другая модель (HL χ^2 = 11,1) показала, что пары «мужчина-мальчик» посещали врачей с большей вероятностью по сравнению с парами «женщина-девочка» (OR 0,23; 95% CI 0,06–0,93; p = 0,039). Не было замечено статистической зависимости между возрастом детей или лиц, осуществляющих уход, местом проведения исследования или ВИЧ-статусом детей (подтверждён или подозревается).

Заключение: В нашей камерунской выборке детей, нуждающихся в последующем ВИЧ-уходе, профиль детей, которые не соблюдают посещения врачей в рамках ВИЧ-программы, является следующим: ребенок женского пола, уход за которым осуществляет лицо без формального образования, и у которого более длительный перерыв между назначенными посещениями врача. Это исследование также показало вероятность того, что женщины предпочитают ухаживать за девочками, а мужчины за мальчиками там, где это касается оказания медицинской помощи.

Translated from English version into Russian by Natalia Potashnik, through

Factores asociados a la inobservancia de las citas de médicas de seguimiento concertadas en niños cameruneses en tratamiento por VIH: un estudio de casos y controles del grupo de tratamiento habitual del ensayo MORE-CARE

Jean Joel R. Bigna, Jean Jacques N. Noubiap, Claudia S. Plottel, Charles Kouanfack, Sinata Koulla-Shiro

Resumen

Antecedentes: Una mejor comprensión de las razones por las cuales los niños expuestos/infectados por el VIH no asisten a sus citas médicas de seguimiento concertadas posibilitaría la intervención para fomentar la prestación de dicha asistencia. El objetivo del presente estudio ha sido establecer las características de la pareja cuidador - niño (CCD) en relación con lainobservancia por parte de este a las citas médicas de seguimiento concertadas en programas de VIH en Camerún.

Métodos: Hemos llevado a cabo un estudio de casos y controles del grupo habitual de tratamiento (control) de CCD procedente del ensayo MORE CARE, en el cual el efecto de los recordatorios en el teléfono móvil para niños expuestos/infectados por VIH sobre la asistencia a las citas de seguimiento ha sido evaluado en tres contextos (urbano, semiurbano y rural), entre los meses de enero y marzo de 2013. En el presente estudio, la falta de un niño a su cita se ha considerado caso y su presencia se ha definido como control. Hemos utilizado un análisis de regresión logística con multivariante de datos binarios. El modelo de ajuste óptimo resultó ser aquel con el menor valor chi cuadrado en el *test Hosmer-Lemeshow* (HL χ^2). Las magnitud de asociación se ha expresado por medio de la oportunidad relativa (OR) con un valor de *p*0,05, considerado estadísticamente significativo.

Resultados: De entre los 30 niños que faltaron a sus citas (casos) y los 31 que asistieron a seguimiento (controles), la edad promedio ha sido de 1,3 (desviación típica 1,9) y 2,0 (2,8) años (p=0,29) respectivamente, y la edad promedio de los cuidadores de 41,9 (13,2) y 43,2 (12,1) años (p = 0,70), respectivamente. Nuestro modelo de ajuste óptimo que ha tenido en cuenta el sexo de adultos y niños por separado (HL χ^2 = 3,5) muestra que la falta de asistencia a las citas médicas concertadas estaba relacionada con: carencia de una educación formal del cuidador (OR 29,1, 95% CI 1,1–777,0; p = 0,044), intervalo de tiempo demasiado largo hasta la próxima cita/seguimiento (OR [incremento 1 semana] 1,4, 95% CI 1,03–2,0; p = 0,032), y la condición de género femenino del niño (OR 5,2, 95% CI 1,2–23,1; p = 0,032). Otros modelos han demostrado la relación entre la condición de niña de la CCD y la inobservancia de las citas, independientemente del género del cuidador. Un modelo de regresión (HL χ^2 = 10,5) ha revelado que las parejas mujer-niño observaban menos las citas médicas que las formadas por mujer-niña (OR 4,9, 95% CI 1,05–22,9; p = 0,044). Otro modelo (HL χ^2 = 11.1) ha revelado que es más probable que parejas hombre-niño asistan a las citas que las formadas por mujer-niña (OR 0,23, 95% CI 0,06-0,93; p = 0,039). No se ha observado relación estadística entre las edades de niños y cuidadores, las ubicaciones del estudio o el estado del VIH (confirmado o presunto) de los niños.

Conclusión: De nuestra muestra camerunesa de niños en seguimiento por VIH, el perfil de aquellos que no asistían a las citas médicas de seguimiento del programa de VIH era: hembra, con cuidador sin educación formal y con mayores intervalos de tiempo entre citas de seguimiento. El estudio también ha mostrado que existe la posibilidad de que las cuidadoras femeninas favorezcan a las niñas y que los cuidadores masculinos favorezcan a los niños.

Translated from English version into Spanish by Raquel Bentué, through
